# Supplementary material for: Estradiol Induces Epithelial to Mesenchymal Transition of Human Glioblastoma Cells
Source: Cells. 2020 Aug 21;9(9):1930. doi: 10.3390/cells9091930 (PMC7564468; doi:10.3390/cells9091930)
Supplement: Supplementary file 1 [file cells-09-01930-s001.pdf]

Estradiol Induces Epithelial to Mesenchymal Transition of Human Glioblastoma Cells

-Supplementary figures-

Ana M. Hernández-Vega[1], Aylin Del Moral-Morales[1], Carmen J. Zamora-Sanchez[1], Ana G. Piña-Medina[2], Aliesha A. González-Arenas[3] and Ignacio Camacho-Arroyo[1*]

1. Unidad de Investigación en Reproducción Humana, Instituto Nacional de Perinatología-Facultad de Química, Universidad Nacional Autónoma de México, CP 04510, CDMX, México
2. Departamento de Biología, Facultad de Química, Universidad Nacional Autónoma de México, CP 04510, CDMX, México
3. Departamento de Medicina Genómica y Toxicología Ambiental, Instituto de Investigaciones Biomédicas, Universidad Nacional Autónoma de México, CP 04510, CDMX, México

*Corresponding author:

Dr. Ignacio Camacho-Arroyo,

Unidad de Investigación en Reproducción Humana,

Instituto Nacional de Perinatología-Facultad de Química, Universidad Nacional Autónoma de México,

Ciudad de México, México;

Tel: (+52) 55 5520 9900/5622 3732; E-mail address: camachoarroyo@gmail.com

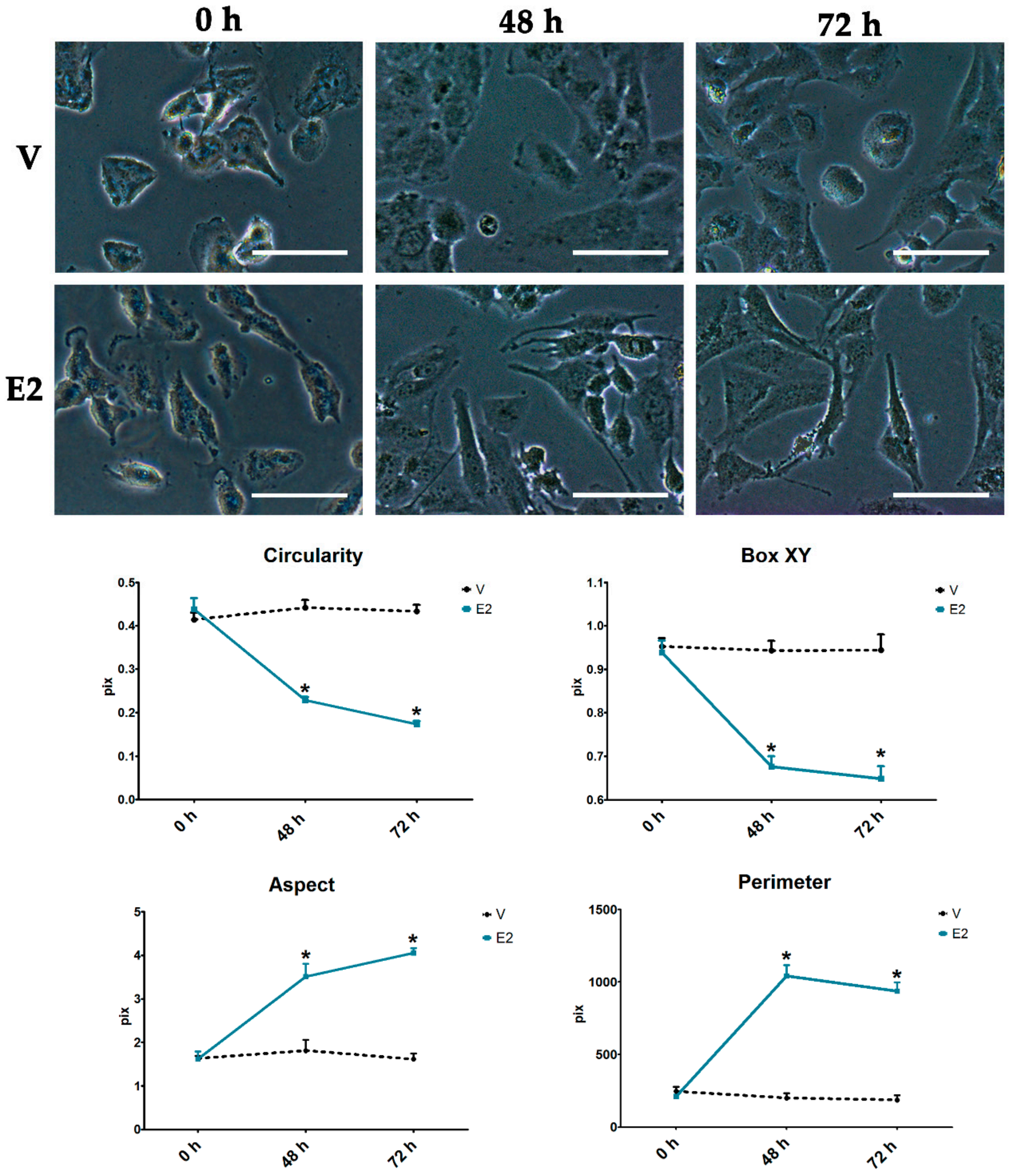

Figure S1. E2-induced morphological changes in human GBM-derived T98G cells. T98G cells observed by phase-contrast microscopy with a magnification of 400X at 0, 48, and 72 hours after adding 17β-estradiol (E2, 10 nM) and the vehicle (V, 0.01% cyclodextrin). Magnification white bar = 100 μm. Plots represent the quantification of

the geometric parameters (circularity, box XY, aspect, perimeter) in this study. Results are expressed as the mean ± standard error of the mean (S.E.M.); n = 3; *p<0.05 vs. V.

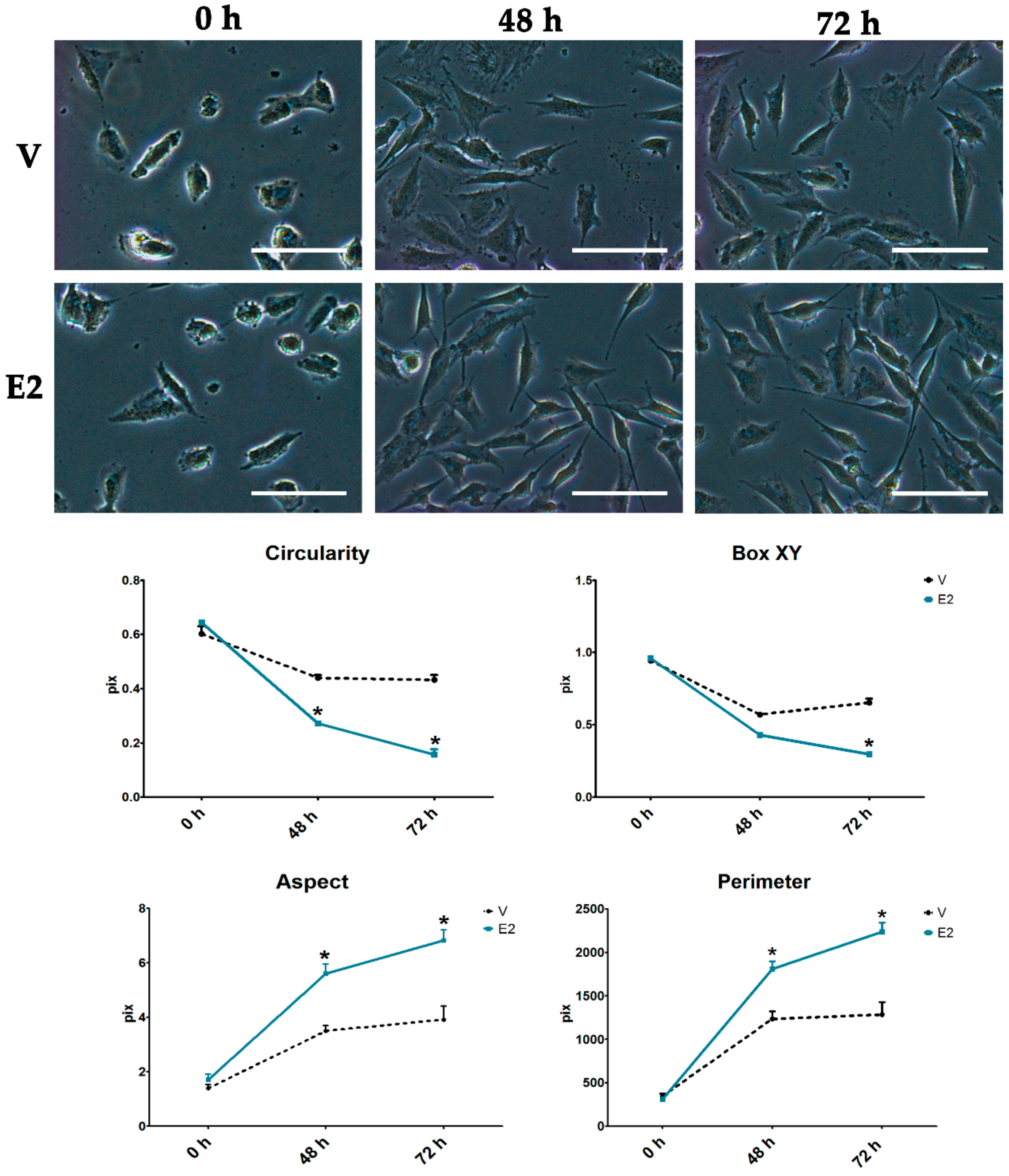

Figure S2. E2-induced morphological changes in human GBM-derived LN229 cells. LN229 cells observed by phase-contrast microscopy with a magnification of 400X at 0, 48, and 72 hours after adding 17β-estradiol (E2, 10 nM) and the vehicle (V, 0.01% cyclodextrin). Magnification white bar = 100 μm. Plots represent the quantification of the geometric parameters (circularity, box XY, aspect, perimeter) in this study. Results are expressed as the mean ± standard error of the mean (S.E.M.); n = 3; *p<0.05 vs. V.
